# Dynamic Severity Assessment of Partial Discharge in HV Bushings Based on the Evolution Characteristics of Dense Clusters in PRPD Patterns

**DOI:** 10.3390/s25247537

**Published:** 2025-12-11

**Authors:** Xiang Gao, Zhiyu Li, Zuoming Xu, Pengbo Yin, Xiongjie Xie, Xiaochen Yang, Baoquan Wan

**Affiliations:** State Key Laboratory of Power Grid Environmental Protection, China Electric Power Research Institute, Wuhan 430074, China; gaoxiang0117@163.com (X.G.); lizhiyu@epri.sgcc.com.cn (Z.L.); xuzuoming@epri.sgcc.com.cn (Z.X.); yinpengbo@epri.sgcc.com.cn (P.Y.); xiexiongjie@epri.sgcc.com.cn (X.X.); sddz2yxc@163.com (X.Y.)

**Keywords:** high-voltage bushing, partial discharge, PRPD pattern, dense discharge cluster, evolution characteristics, severity assessment, fuzzy evaluation

## Abstract

High-voltage bushings are critical insulation components, yet conventional PRPD-based severity assessment methods that rely on global pattern morphologies such as “rabbit ears” and “tortoise shell” remain coarse, lack local sensitivity, and fail to track continuous degradation. This paper proposes a dynamic severity assessment method that shifts the focus from global contours to dense partial discharge (PD) clusters, defined as high-density aggregations of PD pulses in specific phase–magnitude regions of PRPD patterns. Each dense cluster is treated as the statistical projection of a physical discharge channel, and the evolution of its number, intensity, location, and shape provides a fine-scale description of defect development. A multi-level relative density and morphological image processing algorithm is used to extract dense clusters directly from PRPD histograms, followed by a 20-dimensional feature set and a five-index system describing discharge activity, development speed, complexity, instability, and evolution trend. A fuzzy comprehensive evaluation model further converts these indices into three severity levels with confidence measures. Long-term degradation tests on defective bushings demonstrate that the proposed method captures key turning points from dispersed multi-cluster patterns to a single dominant cluster and yields a stable, stage-consistent severity evaluation, offering a more sensitive and physically interpretable tool for condition monitoring and early warning of HV bushings. The method achieved a high evaluation confidence (average 60.1%), which rose to 100% at the critical failure stage. It successfully identified three distinct degradation stages (stable, accelerated, and critical) across the 49 test intervals. A quantitative comparison demonstrated significant advantages: 8.3% improvement in early warning (4 windows earlier than IEC 60270), 50.6% higher monotonicity, 125.2% better stability, and 45.9% wider dynamic range, while maintaining physical interpretability and requiring no training data.

## 1. Introduction

High-voltage (HV) bushings are key insulation components in transmission and transformation equipment, and their insulation condition is directly related to the safe and stable operation of power grids [1,2]. With increasing voltage levels, more compact structures, and higher transmission capacity, local defects inside the condenser core are more likely, under long-term electrical, thermal, mechanical, and electromagnetic stresses, to undergo structural evolution and cause electric field distortion, which, in turn, initiates partial discharge (PD) activity [3,4,5]. If such defects continue to develop, discharge channels gradually extend and may form tree-like paths or penetrating breakdown channels, eventually leading to surface flashover, core rupture, or even explosive failures of the equipment [6,7,8,9]. Engineering practice indicates that bushing insulation degradation usually proceeds through a latent stage, an accelerated stage, and a sudden failure stage. In the early stage, PD signals are random and inconspicuous, whereas once the system enters a critical stage, breakdown development accelerates rapidly in an exponential manner, leaving only a very limited time window for inspection and decision-making on site [10,11]. Therefore, accurately revealing the dynamic evolution of PD from inception to failure, and establishing an assessment model capable of continuously quantifying severity, are crucial for state awareness, degradation tracking, and lifetime prediction of HV equipment.

Phase-resolved partial discharge (PRPD) patterns are an important tool to describe the coupling between PD activity and the electric field, and they contain rich information about defect types, field distortion, and the evolution of discharge channels [12]. Most conventional studies focus on a classification based on global PRPD morphology, summarizing patterns into typical shapes such as “rabbit-ear” or “tortoise-shell” and linking them empirically to specific internal defects [13,14,15,16,17]. Although such methods are useful as references for mechanism understanding and defect identification, they have three fundamental limitations for severity assessment:

1. Coarse representation. The global contour compresses abundant local structures into a single pattern label and is insensitive to subtle changes such as local density redistribution, cluster formation, or intermittent discharges.
2. Insufficient discrimination. Different degradation stages may present similar global PRPD shapes while corresponding to completely different energy levels and failure risks, which can lead to misjudgment of “same shape but different state”.
3. Weak ability to describe continuous evolution. Changes in global morphology are usually discrete and delayed. When an obvious pattern transition is observed, the insulation condition may already be close to critical, making it difficult to support early warning.

To overcome these limitations, this paper shifts the analysis perspective from the global PRPD contour to local high-density regions, referred to as dense PD clusters. A dense PD cluster is defined as a high-density structure formed by a large number of PD pulses statistically concentrated within a limited region of the phase–magnitude plane. From a physical viewpoint, each cluster can be regarded as the statistical projection of a specific discharge channel or a group of channels: the number of clusters reflects the number and competition of discharge sources; the cluster intensity characterizes discharge activity; the cluster location is related to local electric field distribution and defect position; and the cluster shape and its evolution record the expansion, merging, and dominance of discharge channels. Compared with a single global morphology label, dense PD clusters act at a more fundamental physical level. They are able to capture gradual evolution in local microstructures before significant changes appear in the macroscopic pattern, thereby providing a more sensitive and physically meaningful basis for severity assessment.

Dynamic severity assessment based on dense PD clusters involves three key technical challenges. The first is robust and efficient cluster extraction. In practical PRPD data, the volume of pulses is large; background noise and sparse discharges are widely present; clusters may overlap; and density differences occur across multiple scales. Directly applying traditional clustering algorithms to raw pulse clouds is often limited by computational cost, parameter sensitivity, and poor adaptability to different density levels [18,19,20,21,22]. The second is a systematic characterization of cluster properties. Only counting cluster number, maximum apparent charge, or simple geometric measures is insufficient to describe the complex evolution from dispersed multi-source discharges to a concentrated single-source pattern. A multi-dimensional feature system is needed that combines geometric morphology, statistical distribution, periodic structure, and nonlinear complexity, and maps cluster evolution to quantitatively interpretable indicators. The third is a comprehensive evaluation with multiple indices. The 20-dimensional features evolve jointly along the time axis and exhibit correlation and fuzzy transition regions. An appropriate evaluation model is required to map these indices into intuitive severity levels with explicit confidence, while handling uncertainty and gradual boundaries; simple hard thresholds based on empirical settings are inadequate.

To address these challenges, this paper proposes a dynamic severity assessment method for PD in HV bushings based on the evolution characteristics of dense PD clusters in PRPD patterns and establishes an integrated framework from cluster identification and feature extraction to fuzzy comprehensive evaluation. The main contributions are summarized as follows:

1. A dense PD cluster identification algorithm based on multi-level relative density thresholds and morphological image processing is proposed. It operates directly on the two-dimensional PRPD density histogram, achieving fast extraction of clusters with different intensities and shapes while maintaining spatial continuity and strong noise suppression.
2. A comprehensive multi-dimensional feature system is constructed, encompassing geometric morphology, statistical distribution, frequency-domain characteristics, and nonlinear complexity to systematically characterize cluster evolution, including number variation, spatial dynamics, energy concentration, and structural ordering.
3. A multi-perspective severity index system is established, consisting of discharge activity, development speed, complexity, instability, and evolution trend. A fuzzy comprehensive evaluation model with triangular membership functions is introduced to map the time-varying multi-index vector to three severity levels and corresponding confidence, thus quantitatively describing level competition and uncertainty in transition regions.
4. Long-term degradation experiments on defective HV bushings, spanning the entire process from PD inception to critical failure, verify that the proposed method clearly reveals the typical evolution from dispersed multi-cluster patterns to a single dominant cluster, identifies key transition phases that are difficult to detect using conventional global morphology methods, and achieves good consistency between assessment results and the actual failure process, providing a feasible technical approach for condition monitoring and preventive maintenance of HV bushings.

## 2. Methods

### 2.1. Construction of PRPD Patterns and Identification of Dense PD Clusters

#### 2.1.1. Two-Dimensional PRPD Histogram

**Figure 1:**
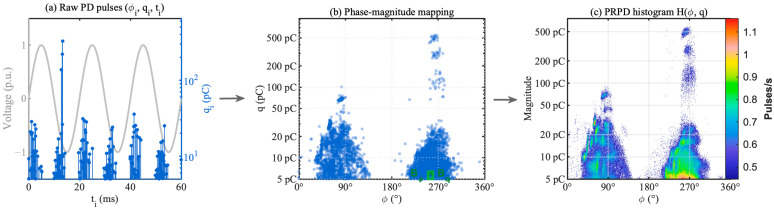
Illustration of PRPD pattern construction: (**a**) raw PD pulses as triplets (φ_i_, q_i_, t_i_) overlaid on power-frequency voltage; (**b**) mapping to phase-magnitude plane; (**c**) normalized PRPD density histogram.

Phase-resolved analysis of PD in HV bushings is based on a two-dimensional PRPD density histogram. Each detected PD pulse is expressed as a triplet $\left(\varphi_{i},q_{i},t_{i}\right)$, where $\varphi_{i}$ is the phase angle of the power-frequency voltage (0–360°), $q_{i}$ is the apparent charge (pC), and $t_{i}$ is the timestamp. To obtain a statistically meaningful PRPD pattern, the continuous phase–magnitude space is discretized into finite bins. Figure 1 illustrates the complete PRPD construction process from raw PD pulses to the final density histogram.

In this work, the phase axis is divided into 500 bins and the magnitude axis into 400 bins. The magnitude axis is defined on a logarithmic scale (${log}_{10}$ from 5 pC to 50,000 pC) to enhance the resolution of low-magnitude regions. The normalized PRPD density histogram $H\left(\phi ,q\right)$ is defined as

(1) $$H\left(\phi ,q\right)=\frac{1}{\Delta t}\sum_{i=1}^{N}1\left[\phi_{i}\in B_{\phi },q_{i}\in B_{q}\right],$$

where $\Delta t=max\left(tᵢ\right)-min\left(tᵢ\right)$ is the observation time, $B_{\phi }$ and $B_{q}$ are the phase and magnitude bin intervals, and $1\left[\cdot \right]$ is the indicator function. This normalization converts discharge counts to discharge rates (pulses/s), allowing results from different test durations to be comparable.

Unlike traditional PRPD analysis that focuses on overall morphology, this study innovatively concentrates on the local high-density regions within the pattern, referred to as dense PD clusters. From a physical perspective, a dense PD cluster corresponds to a specific discharge channel or a group of channels, whose formation originates from the non-uniform distribution of the electric field and the localized characteristics of insulation defects. Mathematically, each cluster $C_{k}$ in the cluster set $C=C_{1},C_{2},\ldots ,C_{K}$ is completely characterized by its contour boundary $\partial C_{k}$, centroid position $\left(\bar{\phi_{k}},\bar{q_{k}}\right)$, total intensity $I_{k}=\sum_{\left(\phi ,q\right)\in C_{k}}H\left(\phi ,q\right)$, and covered area $A_{k}=\left|C_{k}\right|$. Compared with the traditional global morphology-based description, this representation provides richer local information and enables the capture of subtle variations during the insulation degradation process.

#### 2.1.2. Identification of Dense PD Clusters Based on Multi-Level Density Thresholds

Traditional clustering algorithms encounter problems of low computational efficiency and noise sensitivity when processing PRPD density maps. This study proposes a dense PD cluster identification method based on multi-level density thresholds combined with morphological image processing. The method operates directly on the two-dimensional PRPD density histogram, providing higher computational efficiency and better spatial continuity.

To adaptively identify discharge clusters of different density levels, a three-level thresholding scheme is applied to the PRPD density map:

(2) $$\theta_{i}=\alpha_{i}\times \max\left(H\left(\phi ,q\right)\right),i=1,2,3,$$

where $H\left(\phi ,q\right)$ denotes the PRPD density histogram, $\max\left(H\left(\phi ,q\right)\right)$ is the maximum density value, and$\;\alpha_{i}$ are threshold coefficients. In this study, the parameters are set to $\alpha_{1}=0.2$ (high-density layer), $\alpha_{2}=0.1$ (medium-density layer), and $\alpha_{3}=0.05$ (low-density layer), which correspond to the discharge core region, the main active region, and the peripheral diffusion region, respectively. Each layer is binarized as follows:

(3) $$B_{i}(\phi ,q)=\left\{\begin{array}{ll} 1, & H(\phi ,q)>\theta_{i}\\ 0, & H(\phi ,q)\leq \theta_{i} \end{array}\right.$$

To remove noise and refine the cluster boundaries, a sequence of morphological operations is performed on each binary image:

(4) $$B_{i}^{\prime }=Close(Open(B_{i},SE),SE)⊕Fill,$$

where Open(·) represents the opening operation (to remove isolated noise points), Close(·) represents the closing operation (to fill small gaps), SE is a disk-shaped structuring element with a radius of two pixels, and Fill denotes the hole-filling operation.

Independent clusters are then identified using connected-component labeling, from which the boundaries, centroids, and areas of each cluster are extracted. To avoid duplicate detections among layers, the centroid distance between clusters of different levels is calculated as $d_{ij}=\sqrt{{\left(\phi_{c,i}-\phi_{c,j}\right)}^{2}+{\left(q_{c,i}-q_{c,j}\right)}^{2}}$. If $d_{ij}<d_{\min}$ (set to 10 pixels in this paper), the clusters are considered duplicates and merged. Clusters with an area smaller than $A_{\min}$ (set to 5 pixels) are discarded as noise, yielding the final dense-cluster set.

The proposed method has several advantages:

1. High computational efficiency, since it directly processes the 500 × 400 density matrix instead of hundreds of thousands of raw discharge points.
2. Preservation of spatial information, as the phase–magnitude density characteristics are retained.
3. Strong robustness, because the morphological operations effectively suppress noise and boundary fragmentation.
4. Adaptive parameterization, where relative density thresholds automatically adjust to discharge intensity variations at different severity stages.

### 2.2. Comprehensive Feature Extraction Scheme

To comprehensively characterize the multi-scale properties of dense PD clusters, a 20-dimensional feature extraction system is constructed, covering four aspects: geometric and shape features, statistical distribution features, frequency-domain features, and nonlinear features. The feature vector $f={\left[f_{1},f_{2},\ldots ,f_{20}\right]}^{T}$ is designed according to the principles of physical interpretability and mathematical independence. It is important to note that since a single time window often contains multiple discrete clusters (denoted as the set $C=C_{1},C_{2},\ldots ,C_{K}$), the feature vector serves as a statistical descriptor of the entire cluster set. Specifically, geometric features ($f_{1}$–$f_{10}$) are obtained by applying aggregation operators (e.g., summation, averaging, or standard deviation) to the properties of individual clusters, while statistical and nonlinear features ($f_{11}$–$f_{20}$) are computed based on the global density distribution formed by the identified clusters.

#### 2.2.1. Geometric and Shape Features (Dimensions 1–10)

Geometric features describe the macroscopic properties of clusters. The number of clusters $f_{1}=N_{clusters}$ reflects the competition among multiple discharge sources. The total intensity $f_{2}=\sum_{k}I_{k}$ quantifies the overall discharge activity. The mean and standard deviation of cluster intensity are defined as $f_{3}=\bar{I}=\frac{1}{N_{clusters}}\sum_{k}I_{k}$, $f_{4}=\sigma_{I}=\sqrt{\frac{1}{N_{clusters}}\sum_{k}{\left(I_{k}-\bar{I}\right)}^{2}}$, which describe the uniformity of discharge strength distribution. The total area $f_{5}=\sum_{k}A_{k}$ represents the spatial extent of dense discharge regions.

Shape features are derived from the equivalent phase and magnitude widths of clusters. The average phase width and magnitude width are $f_{6}=\bar{W_{\phi }}=\frac{1}{N_{clusters}}\sum_{k}W_{\phi ,k}$, $f_{7}=\bar{W_{q}}=\frac{1}{N_{clusters}}\sum_{k}W_{q,k}$. The aspect ratio $f_{8}=\bar{r_{aspect}}=\bar{W_{\phi }}/\bar{W_{q}}$ reflects the dominant elongation direction of the clusters in the phase–magnitude plane. The symmetry index $f_{9}$ evaluates the degree of approximate 180° symmetry by counting the proportion of symmetric cluster pairs:

(5) $$f_{9}=S_{sym}=\frac{1}{N_{clusters}}\sum_{k}1\left[d_{sym}\left(k\right)<10{}^{\circ}\right],$$

where $d_{sym}\left(k\right)=\underset{j}{\min}\left|\phi_{k}-\left(\phi_{j}+180{}^{\circ}\right)mod\;360{}^{\circ}\right|$ is the minimum phase distance between cluster k and its potential symmetric counterpart. The compactness index $f_{10}=C_{compact}=\frac{1}{N_{clusters}}\sum_{k}\frac{A_{k}}{W_{\phi ,k}\times W_{q,k}}$ quantifies the regularity of cluster shapes. Higher values indicate more compact and uniform cluster geometries.

#### 2.2.2. Statistical Distribution Features (Dimensions 11–15)

Statistical distribution features reveal the overall distribution characteristics of discharges through weighted higher-order moment analysis. For the phase and magnitude distributions, the weights are defined as $w_{i}=\sum_{j}H\left(i,j\right)/\sum H$, $v_{j}=\sum_{i}H\left(i,j\right)/\sum H$. The n-th central moment of the phase distribution is

(6) $$\mu_{\phi }^{\left(n\right)}=\sum_{i}w_{i}{\left(\phi_{i}-\bar{\phi }\right)}^{n},$$

where $\bar{\phi }=\sum_{i}w_{i}\phi_{i}$ is the weighted mean phase. Based on these moments, the phase skewness and kurtosis are calculated as $f_{11}=\gamma_{\phi }=\mu_{\phi }^{\left(3\right)}/{\left(\mu_{\phi }^{\left(2\right)}\right)}^{3/2}$, $f_{12}=\kappa_{\phi }=\mu_{\phi }^{\left(4\right)}/{\left(\mu_{\phi }^{\left(2\right)}\right)}^{2}$. The skewness describes the asymmetry of the phase distribution, while the kurtosis measures its sharpness. Similarly, the magnitude distribution is analyzed in the logarithmic domain. Its central moment is defined as

(7) $$\mu_{q}^{\left(n\right)}=\sum_{j}v_{j}{\left(\log_{10}q_{j}-\bar{\log_{10}q}\right)}^{n},$$

from which the magnitude skewness and kurtosis are obtained as $f_{13}=\gamma_{q}=\mu_{q}^{\left(3\right)}/{\left(\mu_{q}^{\left(2\right)}\right)}^{3/2}$, $f_{14}=\kappa_{q}=\mu_{q}^{\left(4\right)}/{\left(\mu_{q}^{\left(2\right)}\right)}^{2}$. The phase–magnitude correlation coefficient $f_{15}=\rho_{\phi q}=\mathrm{corr}\left(\phi \cdot H,q\cdot H\right)$ characterizes the coupling between phase and magnitude. A high correlation indicates that the discharge activity tends to concentrate at specific phase–magnitude combinations.

#### 2.2.3. Frequency-Domain and Nonlinear Features (Dimensions 16–20)

Frequency-domain features are used to analyze the periodicity of discharge activity. The phase profile $P_{\phi }=\sum_{q}H\left(\phi ,q\right)$ is transformed using the fast Fourier transform (FFT) to obtain the amplitude spectrum:

(8) $$F_{k}={\left|\mathrm{FFT}\left(P_{\phi }\right)\right|}_{k},\;k=1,2,\ldots ,N_{phase}$$

Three frequency-domain features are extracted: $f_{16}=\frac{1}{9}\sum_{k=2}^{10}F_{k}$, $f_{17}=\underset{k>1}{\max}F_{k}$, $f_{18}=\arg\underset{k>1}{\max}F_{k}$. Here, $f_{16}$ represents the mean amplitude of low-frequency harmonics that describe basic discharge patterns, $f_{17}$ represents the maximum spectral component indicating the dominant frequency intensity, and $f_{18}$ gives the index of the dominant frequency component corresponding to the main discharge periodicity.

Nonlinear features quantify the complexity of discharge evolution. The information entropy $f_{19}=S_{entropy}$ measures the degree of disorder of the discharge distribution. The fractal dimension $f_{20}$ is computed using the box-counting method. For box sizes r ∈{2, 4, 8, 16, 32}, the number of boxes required to cover the binarized PRPD map is denoted as N(r). The fractal dimension is estimated by the logarithmic linear fitting:

(9) $$f_{20}=D_{f}=-\frac{\partial \log N\left(r\right)}{\partial \log r}$$

The fractal dimension is constrained within the range [1, 2]. Values close to 1 indicate simple line-like structures, while values close to 2 indicate complex space-filling discharge patterns.

### 2.3. Five-Dimensional Severity Index System

The assessment of insulation severity requires a multi-perspective evaluation of its condition. In this study, a five-dimensional severity index system is established, covering the current state, development speed, system complexity, stability, and evolutionary trend. This framework comprehensively utilizes the extracted 20-dimensional feature information to quantify the overall degradation state of the insulation.

#### 2.3.1. Discharge Activity Index

The discharge activity index reflects the intensity of discharge at the current time relative to the reference state:

(10) $$I_{activity}=\frac{1}{2}\left(\frac{Q_{total}}{Q_{ref}}+\frac{N_{bands}}{N_{ref}}\right),$$

where $Q_{total}=f_{2}$ represents the total discharge intensity, and $N_{bands}=f_{1}$ denotes the number of dense clusters. The reference values $Q_{ref}$ and $N_{ref}$ are calculated as the mean of the first three time windows, or of all available windows if fewer than three exist. This index directly utilizes features $f_{1}$ and $f_{2}$, providing an intuitive measure of the overall discharge activity level.

#### 2.3.2. Development Speed Index

The development speed index captures the rate of evolution of the system based on the temporal variation in the 20-dimensional feature vector:

(11) $$I_{speed}\left(t\right)=\lambda \cdot \left|\left|f_{t}-f_{t-1}\right|\right|+\left(1-\lambda \right)\cdot I_{speed}\left(t-1\right),$$

where $\left|\left|f_{t}-f_{t-1}\right|\right|=\sqrt{\sum_{i=1}^{20}{\left(f_{i,t}-f_{i,t-1}\right)}^{2}}$ is the Euclidean distance between two consecutive feature vectors. The forgetting factor λ= 0.3 ensures that the index emphasizes recent changes while smoothing short-term fluctuations. This exponentially weighted moving average reduces the influence of random variations in single measurements and provides a stable representation of evolution speed.

#### 2.3.3. Complexity Index

The complexity index integrates information entropy and fractal dimension to evaluate the system’s degree of order and structural richness:

(12) $$I_{complexity}=0.6\cdot \frac{f_{19}}{\log_{2}\left(N_{phase}\times N_{mag}\right)}+0.4\cdot \frac{f_{20}-1}{1},$$

where $f_{19}$ is the information entropy and $f_{20}$ is the fractal dimension. Both quantities are normalized to ensure comparability between their scales. A higher complexity index indicates chaotic multi-source activity, while a lower value represents an ordered discharge process dominated by a single source.

#### 2.3.4. Instability Index

The instability index evaluates the predictability of the system by measuring the variance of the 20-dimensional feature vectors within a sliding time window:

(13) $$I_{instability}=\sqrt{\frac{1}{W}\sum_{i=t-W+1}^{t}{\left|\left|f_{i}-\bar{f}\right|\right|}^{2}},$$

where $\bar{f}=\frac{1}{W}\sum_{i=t-W+1}^{t}f_{i}$ is the mean feature vector within the window, and the window size is set to W = 5. This index considers all 20 features to evaluate temporal stability. A high instability value indicates strong short-term fluctuations and may signal the approach of sudden degradation events.

#### 2.3.5. Evolution Trend Index

The evolution trend index estimates the long-term degradation tendency based on the statistical behavior of active trajectories:

(14) $$I_{trend}=\frac{1}{2}\left(\frac{N_{trajectories}}{N_{traj,ref}}+\frac{\bar{I_{evolution}}}{\bar{I_{evo,ref}}}\right),$$

where $N_{trajectories}$ denotes the number of currently active evolutionary trajectories, and $\bar{I_{evolution}}=I_{speed}$ represents the mean evolution intensity. The reference values $N_{traj,ref}$ and $\bar{I_{evo,ref}}$ are obtained from the initial time windows. An increasing trend of $I_{trend}\;$indicates the accumulation of progressive degradation, suggesting that the insulation system is moving toward a more critical state.

### 2.4. Fuzzy Comprehensive Evaluation Method

#### 2.4.1. Design of Fuzzy Membership Functions

The quantitative assessment of severity is performed using a fuzzy comprehensive evaluation method. Three severity levels are defined as V={Level 1 (low), Level 2 (medium), Level 3 (high)}. Each level is represented by a triangular membership function μ(x) that defines its fuzzy boundary:

(15) $$\mu (x)=\left\{\begin{array}{ll} 0, & x\leq a\\ (x-a)/(b-a), & a<x\leq b\\ (c-x)/(c-b), & b<x\leq c\\ 0, & x>c \end{array}\right.,$$

where x is the normalized index value and $\left(a,\;b,c\right)\;$are the parameters of the triangular function. For the five normalized indices in the range [0, 1], the same parameter set is applied to all three severity levels as shown in Table 1.

The boundary values (a, b, c) were determined empirically based on the normalized distribution of severity indices. By normalizing all five indices to the [0, 1] range, the method mitigates the influence of absolute numerical variations caused by different test conditions, representing relative severity progression rather than rigid absolute thresholds. The overlapping design of adjacent levels (e.g., Level 1 and Level 2 overlap in [0.2, 0.4]) reflects the continuous nature of insulation degradation in physical systems. While the current parameters rely on expert experience, this is consistent with established practices where fuzzy logic inference is based on heuristic knowledge of PD physics. Future work will explore data-driven optimization to further refine these boundaries.

#### 2.4.2. Fuzzy Comprehensive Evaluation and Confidence Assessment

The fuzzy comprehensive evaluation (FCE) establishes a nonlinear mapping from the five severity indices to the three severity levels.

The factor set U is composed of the five severity indices defined in Section 2.3: $U=\{I_{activity},I_{speed},I_{complexity},I_{instability},I_{trend}\}$. The evaluation set V corresponds to the three severity levels defined above: V={Level 1, Level 2, Level 3}.

For any time window ttt, the five-dimensional index vector is $I_{t}={\left[I_{activity},I_{speed},I_{complexity},I_{instability},I_{trend}\right]}_{t}={\left[I_{1},I_{2},I_{3},I_{4},I_{5}\right]}_{t}$. After normalization, each normalized index $I_{norm,t}$ is substituted into the three membership functions defined in Table 1 to obtain its membership degrees $\mu_{ij}=\mu_{\mathrm{Level}\;j}\left(I_{i,norm}\right)$. This yields a 5×3 fuzzy relation matrix R:

(16) $$R=\left(\begin{array}{ccc} \mu_{11} & \mu_{12} & \mu_{13}\\ \mu_{21} & \mu_{22} & \mu_{23}\\ \mu_{31} & \mu_{32} & \mu_{33}\\ \mu_{41} & \mu_{42} & \mu_{43}\\ \mu_{51} & \mu_{52} & \mu_{53} \end{array}\right),$$

where $\mu_{ij}$ denotes the membership degree of the i-th index to the j-th severity level.

Since the five indices contribute unequally to the final severity level, the analytic hierarchy process (AHP) is used to determine the weight vector $W=\left[w_{1},w_{2},w_{3},w_{4},w_{5}\right]=\left[0.25,0.2,0.2,0.2,0.15\right]$. The weights emphasize the importance of the discharge activity index (0.25) and assign auxiliary weight to the evolution trend (0.15).

A weighted average operator ($M\left(\cdot ,+\right)$) is used for fuzzy synthesis, resulting in the comprehensive evaluation vector

(17) $$B=W\circ R=\left[b_{1},b_{2},b_{3}\right],$$

where $b_{j}=\sum_{i=1}^{5}\;w_{i}\cdot \mu_{ij},j=1,2,3$.

According to the principle of maximum membership, the final severity level is determined as $L=\arg\underset{j}{\max}\left(b_{j}\right)$, which corresponds to the severity level with the highest membership value in vector B.

To quantify the clarity of the evaluation, the confidence index $C_{confidence}$ is introduced based on information entropy. After normalization $P=B/\sum b_{j}$, the entropy is calculated as

(18) $$E=-\sum_{j=1}^{3}p_{j}\log_{2}\left(p_{j}\right),$$

where the maximum entropy $E_{max}=\log_{2}\left(3\right)$ corresponds to a completely uncertain state in which all three membership values are equal. The confidence is defined as

(19) $$C_{confidence}=1-\frac{E}{E_{max}},$$

A confidence value close to 1 indicates that the evaluation result is highly concentrated on a single level, representing a clear and reliable judgment. A confidence close to 0 implies that the membership degrees are evenly distributed among the levels, suggesting that the system is in a transitional state with high uncertainty.

## 3. Experimental Verification and Result Analysis

### 3.1. Preparation of Defective Bushing Models

**Figure 2:**
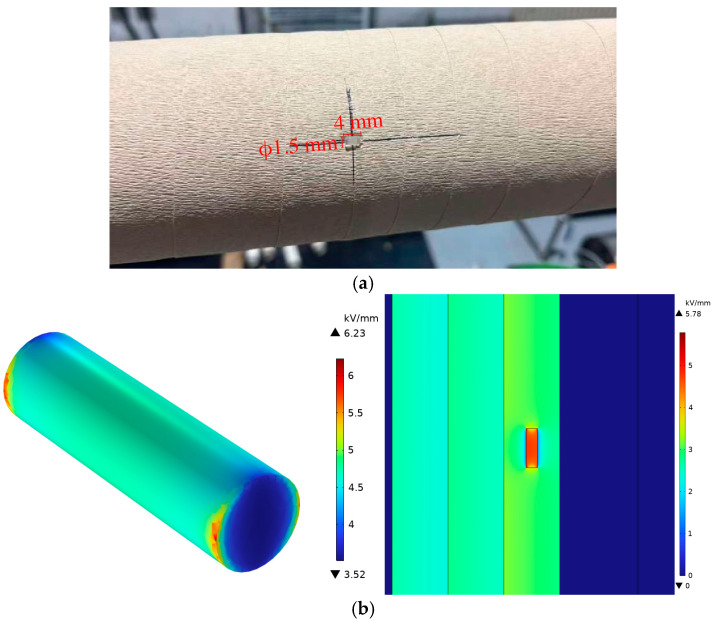
Simulated internal-void defect bushing core: (**a**) photograph captured during winding process with dimension annotations; (**b**) electric-field simulation results showing the isolated void (**left**) and cross-sectional view within the condenser core (**right**).

The experiment employed a simulated internal-void defect model based on an epoxy-impregnated paper (ECAA-40.5/630-4) capacitive bushing. The condenser core of the bushing was designed with a diameter of 110 mm and four electrode layers. As shown in Figure 2a, during the core-winding process, an artificial void encapsulated in an epoxy block was inserted between the second and third electrode layers within the insulation layer. The photograph was taken before the outer insulation layers were wrapped, and the void became entirely internal after the winding process was completed. The void had a diameter of 1.5 mm and a height of 4 mm. Figure 2b presents the simulated electric-field distribution: the left panel shows the 3D field distribution of the isolated void (maximum: 6.23 kV/mm), and the right panel shows the cross-sectional view of the void embedded within the bushing condenser core (maximum: 5.78 kV/mm). Under a peak voltage of 40.5 kV, both maximum field strengths exceed the breakdown field strength of the gas medium.

**Figure 3:**
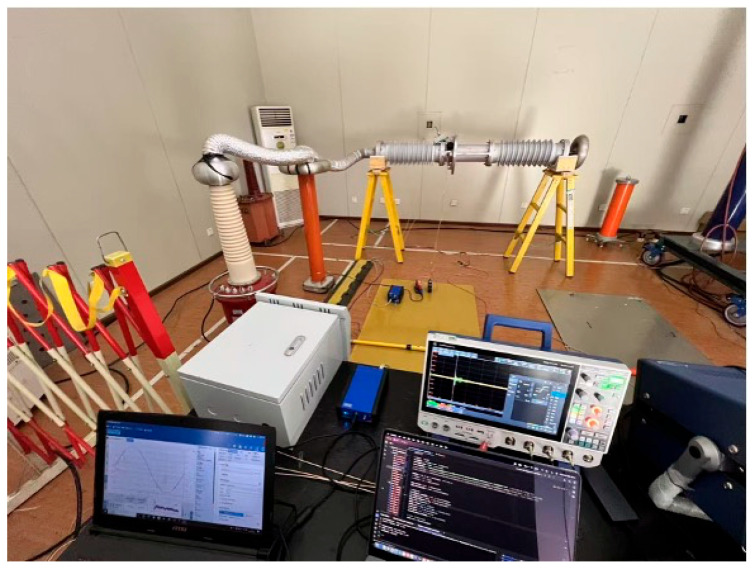
Partial-discharge test platform for bushing samples.

The experimental platform is illustrated in Figure 3. The test setup consisted of a 150 kV PD-free power-frequency test transformer. The bushing sample was supported by insulating ladders to ensure electrical isolation from the ground. Aluminum foil corrugated tubes were employed at high-voltage connection points to suppress corona interference and minimize background noise. A capacitive voltage divider was connected in parallel with the bushing to measure the applied voltage and provide the phase synchronization signal for PRPD analysis. An Omicron MCT 120 High Frequency Current Transformer (HFCT; OMICRON electronics GmbH, Klaus, Austria) was installed on the bushing test tap grounding lead to capture PD signals. The HFCT has a frequency bandwidth of 80 kHz to 40 MHz (−6 dB). The signals were acquired using an Omicron MPD 800 digital PD measurement system (OMICRON electronics GmbH, Klaus, Austria) with a sampling rate of 125 MS/s.

First, a 40.5 kV power-frequency voltage was applied to the normal bushing, and the measured apparent discharge was less than 3 pC, confirming that the test circuit was free from external interference. Subsequently, the defective bushing samples were subjected to insulation degradation tests. The PRPD patterns were recorded continuously, capturing the dynamic evolution of partial-discharge activity from inception to severe failure.

### 3.2. Dense PD Cluster Identification and Evolution Analysis

#### 3.2.1. Data Acquisition and Processing

From the inception of partial discharge to the stage of severe failure, a total of 49 time windows were recorded. The experiment adopted a combined voltage application strategy: the voltage was first stepped up from the partial discharge inception voltage (PDIV) of 18 kV to 40 kV with an increment of 1 kV per minute (Windows 1–23) and then maintained at 40 kV until the final breakdown (Windows 24–49). Each time window had a duration of 60 s. The number of detected PD events exhibited distinct growth phases. During windows 1–7, the number of pulses increased from 35,000 to 55,000. During windows 8–20, it rose rapidly to 428,000. During windows 21–27, the count reached a peak of 3.32 million before dropping to 710,000. During windows 28–43, it remained between 700,000 and 900,000. Finally, during windows 44–49, the discharge quantity increased sharply, reaching 5.3 million in the last window, which was more than 150 times the initial level.

#### 3.2.2. Results of Dense PD Cluster Identification

**Figure 4:**
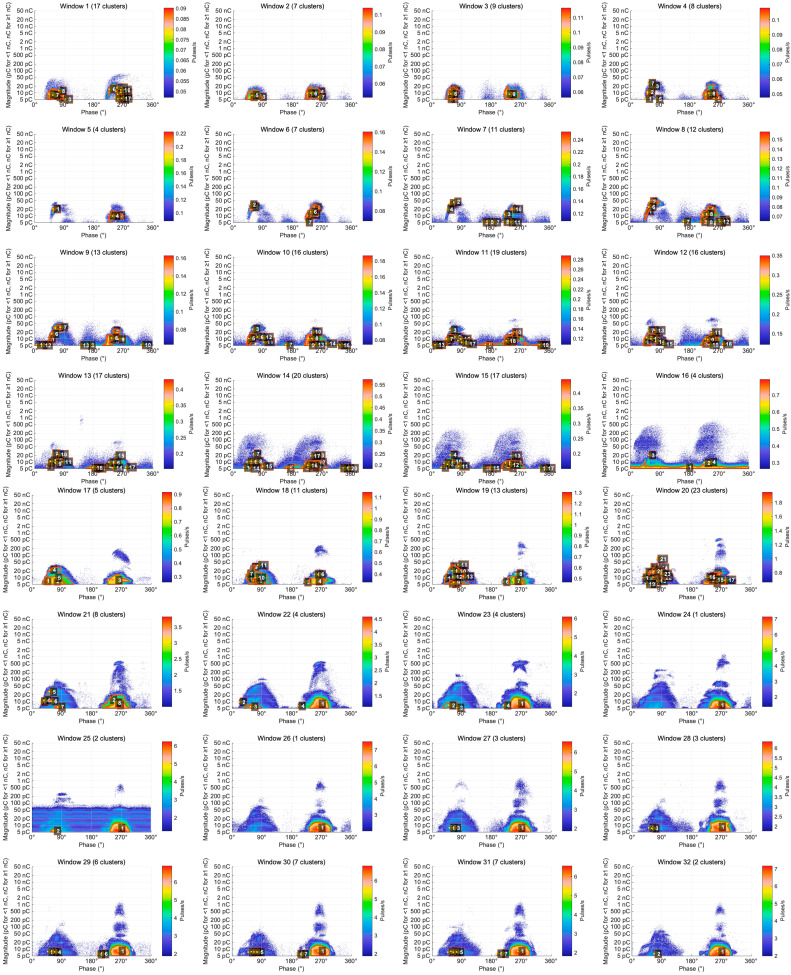

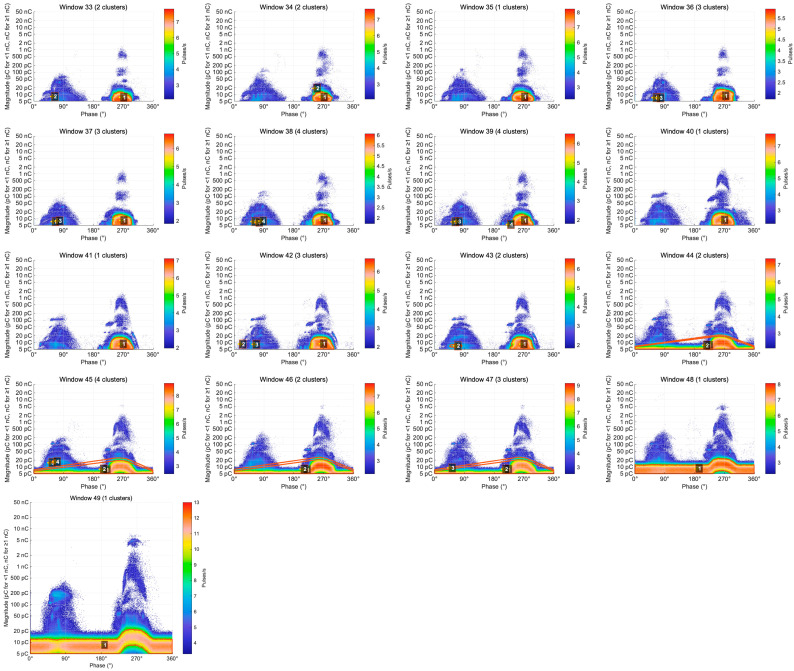
Evolution of dense PD clusters across 49 time windows under a constant applied voltage of 40.5 kV. Each time window represents a data acquisition duration of 60 s. The white numbers inside the black squares represent the unique Cluster IDs assigned by the tracking algorithm to identify specific discharge sources across the sequence.

Dense PD clusters were successfully identified across all 49 time windows. As illustrated in Figure 4, each cluster is visualized using a fine orange–red contour and labeled with a white number inside a black square (representing the unique Cluster ID assigned by the tracking algorithm), indicating its spatial distribution and temporal evolution trajectory.

The evolution process can be divided into five distinct stages:

1. Initial dispersion stage (windows 1–15): 4–20 clusters were identified, with 17 clusters in window 1 and a local peak of 20 clusters in window 14.
2. Rapid growth stage (windows 16–20): The number of clusters increased sharply from 4 to the overall peak of 23.
3. Merging and contraction stage (windows 21–27): The cluster count dropped rapidly from 8 to 1, with only a single cluster remaining in window 24.
4. Stable development stage (windows 28–43): The number fluctuated between 1 and 7, averaging about 3 clusters per window.
5. Critical burst stage (windows 44–49): The system rapidly converged to 1–3 clusters, with only one giant cluster persisting in the final windows 48 and 49.

Key evolutionary events can be summarized as follows. The cluster-count peak at window 20 (23 clusters) corresponds to the maximum spatial diffusion of discharge activity. The rapid merging between windows 21 and 24 (from 23 to 1 cluster) reflects a phase transition from dispersed low-intensity discharges to a concentrated high-intensity regime. The final convergence observed in windows 48–49 marks the system’s entry into an irreversible critical breakdown state.

### 3.3. Analysis of the 20-Dimensional Feature Evolution

**Figure 5:**
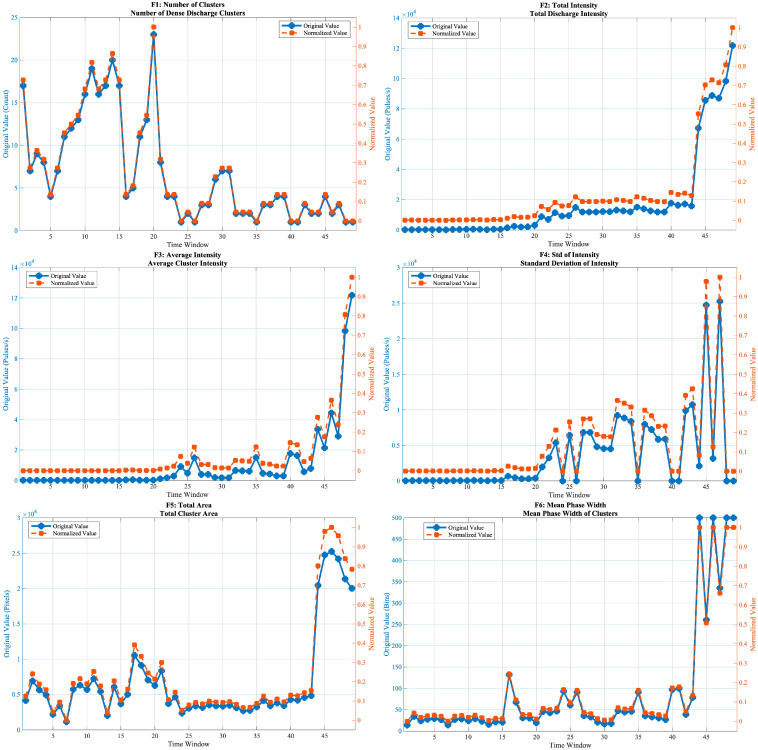

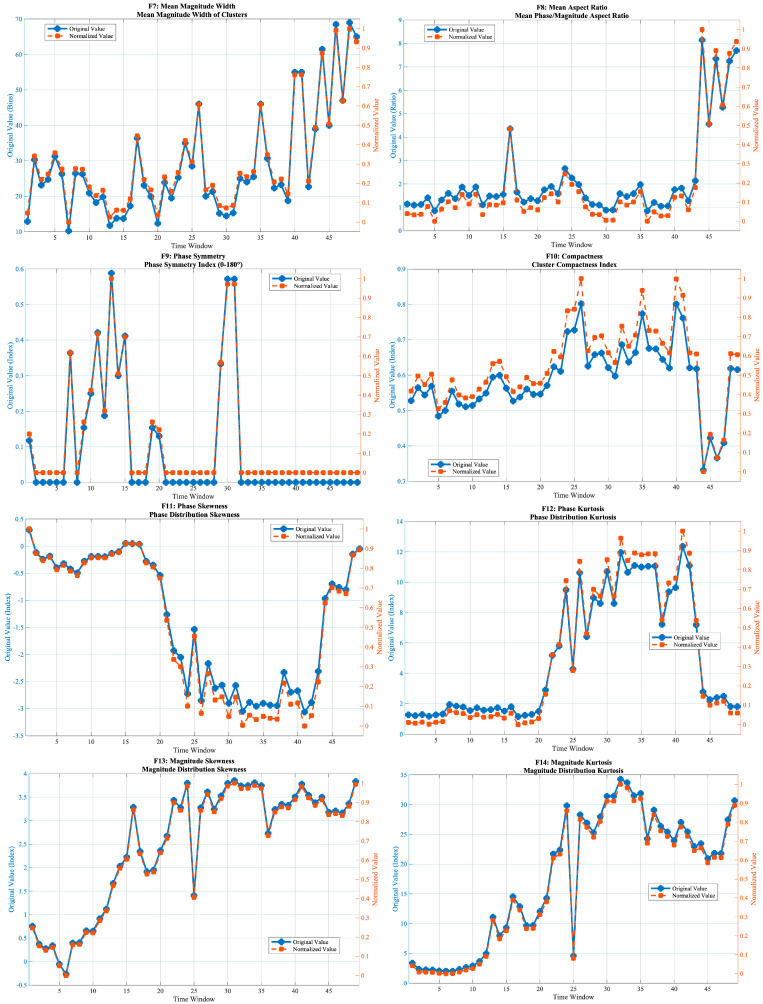

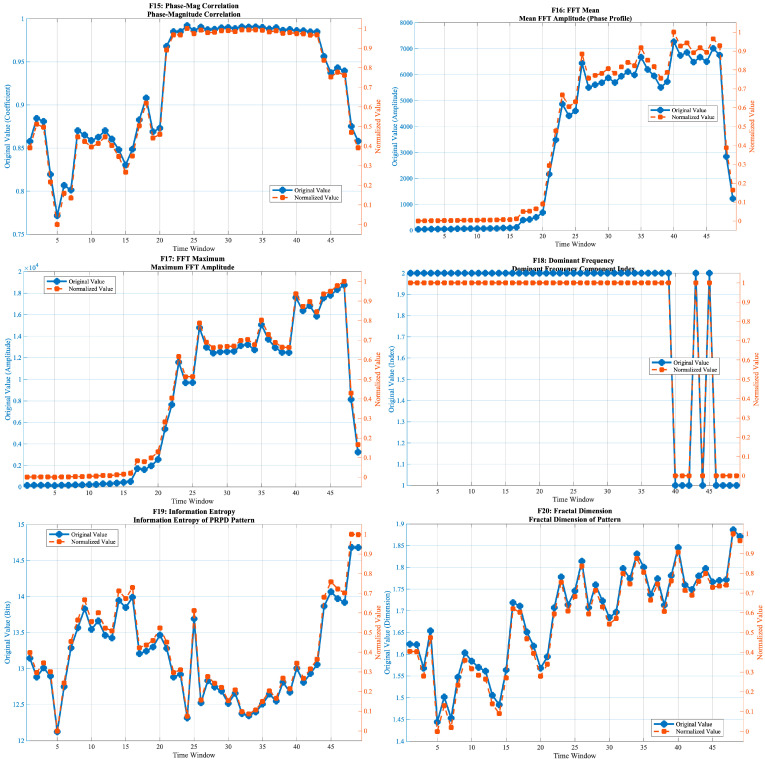
Evolution trajectories of the 20-dimensional feature set across 49 time windows.

Figure 5 presents the normalized evolution curves of all 20 features across the 49 time windows. The results show distinct stage-dependent evolution patterns, with different categories of features exhibiting diverse temporal behaviors.

#### 3.3.1. Morphological Features

The number of discharge clusters ($f_{1}$) shows a continuous decreasing trend, gradually reducing from a high initial value to nearly 1, indicating a transformation from a multi-cluster dispersed discharge mode to a single-cluster concentrated pattern. The total discharge intensity ($f_{2}$) and mean intensity ($f_{3}$) increase monotonically, with a rapid rise in the later stage, reflecting the cumulative growth of discharge energy. The standard deviation of cluster intensity ($f_{3}$) remains close to zero in the early stage, indicating that the discharge energy is relatively evenly distributed among clusters. Starting from the middle stage, f4 exhibits pronounced peaks and fluctuations, which correspond to the emergence and strengthening of dominant clusters and to repeated redistribution of discharge energy. In the final windows, the sharp increase of $f_{4}$ indicates extreme heterogeneity of intensity, consistent with the formation of a single strongly dominant cluster. The total discharge area ($f_{5}$) remains relatively stable in the early phase and expands rapidly after window 40. Both the phase width ($f_{6}$) and amplitude width ($f_{7}$) increase steadily, and their evolution curves are nearly synchronized. The aspect ratio ($f_{8}$) exhibits a distinct jump after window 40, while the phase symmetry ($f_{9}$) drops sharply toward 0 around window 30, suggesting the loss of bipolar symmetry in the PRPD pattern. The compactness ($f_{10}$) rises moderately in the middle stage and fluctuates slightly toward the end.

#### 3.3.2. Statistical Distribution Features

The phase skewness ($f_{11}$) decreases gradually, with a marked drop around window 20. The phase kurtosis ($f_{12}$) rises abruptly near window 25 and continues increasing thereafter. The amplitude skewness ($f_{13}$) and amplitude kurtosis ($f_{14}$) exhibit nearly identical trajectories, both showing a sharp change around window 25, followed by a sustained increase and saturation in the later stage. The phase-amplitude correlation coefficient ($f_{15}$) rises rapidly near window 20 and then stabilizes at a high level, indicating strengthened coupling between discharge phase and magnitude.

#### 3.3.3. Frequency-Domain and Nonlinear Complexity Features

The mean FFT amplitude ($f_{16}$) and the maximum FFT amplitude ($f_{17}$) evolve almost identically, both increasing monotonically and accelerating in the mid-to-late stages. The dominant frequency ($f_{18}$), representing the index of the strongest frequency component in the phase-distribution spectrum, reflects the periodicity of discharge activity. Around window 40, $f_{18}$ drops from 2 to 1, indicating a shift in the dominant spectral component from higher to lower frequency, which corresponds to a transition of the discharge behavior from rapid oscillation to slower periodic variation. The information entropy ($f_{19}$) exhibits a “V-shaped” pattern—first decreasing and then increasing—reaching its minimum near window 25. The fractal dimension ($f_{20}$) rises smoothly and monotonically, suggesting increasing spatial complexity of discharge activity.

### 3.4. Dynamic Severity Assessment

#### 3.4.1. Evolution of the Five-Dimensional Severity Indices

**Figure 6:**
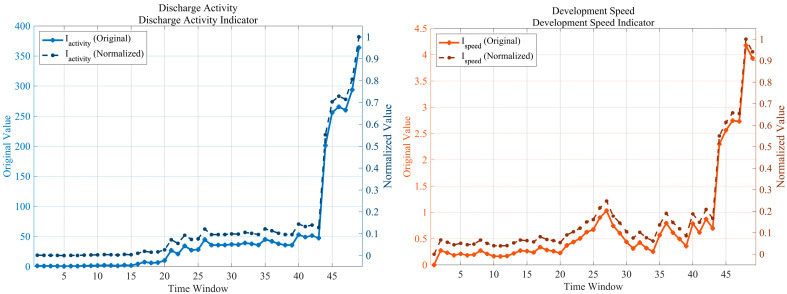

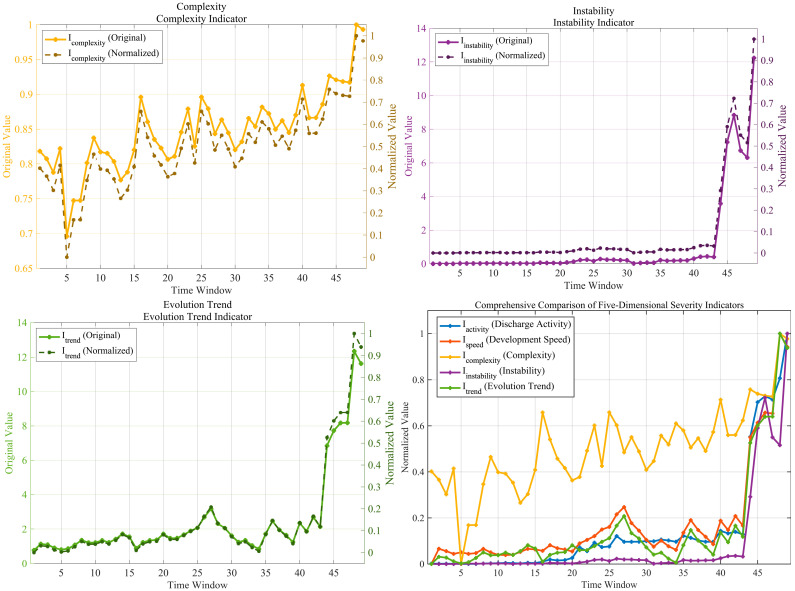
Evolution of the five severity indices derived from the 20-dimensional feature set.

Five severity indices were derived from the 20-dimensional feature set, as illustrated in Figure 6. The figure contains five subplots corresponding to the indices Discharge Activity, Development Speed, Complexity, Instability, and Evolution Trend, each showing both the original and normalized evolution curves. An additional composite subplot compares the normalized trajectories of all five indices.

1. Discharge Activity Index ($I_{activity}$): From windows 1–17, the index remained at a very low level (original 8.53–12.39; normalized 0.0002–0.0046). Between windows 18–43, it rose slowly (15.43–50.19; 0.0021–0.1270), and during windows 44–49, it exhibited an exponential burst (245.9–395.2; 0.5450–1.0000). The peak value of 395.2 was observed at window 49 (normalized 1.0000). The cumulative increase over the first 88% of the duration accounted for only 0.127, while the final 12% contributed 0.873, showing a typical delayed-explosive behavior.
2. Development Speed Index ($I_{speed}$ ): The index fluctuated slightly during windows 1–17 (original 0.14–2.79; normalized 0.0003–0.0618). It experienced a first surge in windows 18–30 (2.14–6.74; 0.0475–0.1496) and a second increase in windows 31–43 (2.45–8.67; 0.0542–0.1921). A critical burst occurred in windows 44–49 (24.58–45.13; 0.5447–1.0000). The first local peak (6.74; normalized 0.1496) appeared at window 25, while the global maximum (45.13; 1.0000) occurred at window 48, followed by a slight decline at window 49 (42.17; 0.9347).
3. Complexity Index ($I_{complexity}$): Windows 1–4 showed moderate complexity (0.79–0.82; 0.3107–0.4029), while window 5 exhibited an abnormally low value (0.67; 0.0056). From windows 6–43, the index remained at a high-level fluctuation range (0.76–0.90; 0.2671–0.7185), and then sharply increased during windows 44–49 (0.87–0.97; 0.5549–1.0000). The maximum of 0.97 was reached at window 48 (normalized 1.0000), followed by a slight drop to 0.96 (0.9704) at window 49. The index remained at a relatively high level (0.4–0.7) through most of the degradation process and exceeded 1.0 at the critical stage, indicating the emergence of a highly complex discharge system.
4. Instability Index ($I_{instability}$): From windows 1–43, the index was nearly zero (0.01–0.93; 0.0001–0.0215). A vertical surge occurred in windows 44–49 (12.89–56.09; 0.2979–1.0000). A stage peak (31.52; 0.7288) was recorded at window 46, followed by a temporary decrease at windows 47–48 (23.55–21.86; 0.5449–0.5055) and a final extreme instability at window 49 (56.09; 1.0000). The index remained below 0.022 for 88% of the total duration and rose from 0 to 1 within the last six windows, representing the steepest growth slope among all indices.
5. Evolution Trend Index ($I_{trend}$): A gradual increase was observed in windows 1–17 (0.27–0.54; 0.0193–0.0392), followed by the first activation phase in windows 18–30 (0.54–2.76; 0.0392–0.2010). The plateau accumulation stage occurred during windows 31–43 (0.70–2.31; 0.0510–0.1684). In windows 44–49, the index exhibited an accelerated climb (7.13–13.73; 0.5190–1.0000). The first local maximum (2.76; 0.2010) appeared at window 27, while the global maximum (13.73; 1.0000) occurred at window 48, followed by a slight decline to 12.75 (0.9286) at window 49.
6. Synergistic behavior of the five indices: A first synergistic peak occurred between windows 25–27, where the development-speed (0.1496–0.1667), evolution-trend (0.1628–0.2010), and discharge-activity indices (first exceeding 0.1) rose simultaneously, corresponding to the merging of dense PD clusters. The second synergistic peak appeared in windows 44–49, where all five indices simultaneously exceeded 0.5, with a mean value of 0.905 at window 48. The interval between the two peaks spanned 17 windows, and the variance among the indices decreased from 0.142 (average during windows 1–43) to 0.082 (windows 44–49) and further to 0.029 at window 48, confirming multi-dimensional convergence at the critical failure stage.

#### 3.4.2. Fuzzy Comprehensive Evaluation Results and Analysis

**Figure 7:**
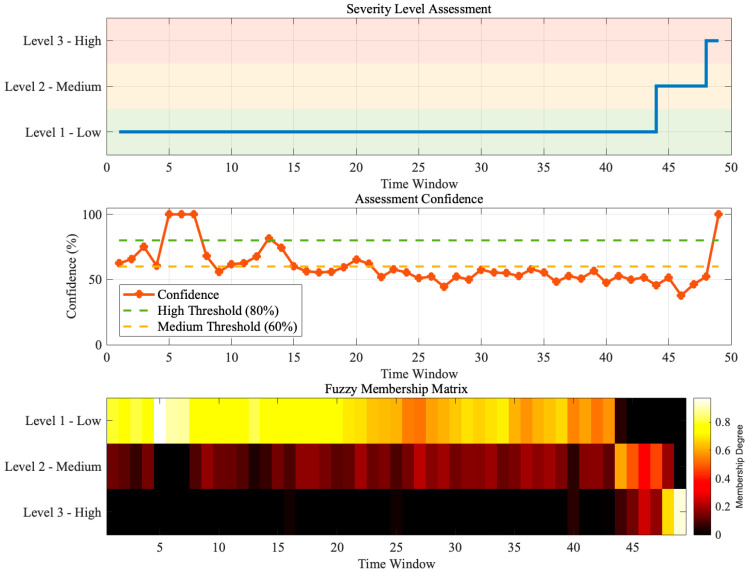
Dynamic fuzzy comprehensive evaluation results based on the five-dimensional severity indices.

Based on the five severity indices, a fuzzy comprehensive evaluation was performed for all 49 time windows, and the results are shown in Figure 7. The figure contains three subplots that, respectively, illustrate (a) the time-series evolution of severity levels, (b) the variation in evaluation confidence, and (c) the temporal evolution of the fuzzy membership matrix.

The evolution of severity levels exhibits a clear stepwise ascending trend. In the first 43 windows (1–43), the evaluation results were all Level 1 (low severity), indicating that the insulation system remained in the early degradation stage. Although feature variations were already observable, the overall risk level was still controllable. During windows 44–46, the severity level increased to Level 2 (moderate severity), marking the onset of accelerated degradation. In windows 47–49, the severity further rose to Level 3 (high severity), implying that the insulation system had approached a critical failure state. This stair-step evolution pattern conforms to the gradual-to-abrupt nature of insulation degradation and aligns well with engineering observations.

The evaluation confidence remained generally high, with values ranging from 37.8% to 100% and an overall mean of 60.1%. In the early stage (windows 1–10), confidence fluctuated significantly, occasionally dropping below the 60% threshold, which indicates weak and unstable degradation signatures. During the middle stage (windows 11–40), the confidence stabilized around 55–65%, showing consistent reliability in the evaluation results. In the transition windows (44 and 47), where the severity level shifted from Level 1 to Level 2 and then to Level 3, temporary confidence drops were observed due to intensified competition among membership grades. In the final stage, confidence rapidly increased, reaching 100% at window 49, signifying that Level 3 features had fully dominated. Overall, the confidence distribution shows a stable central tendency (median 55.9%) and low dispersion (σ = 0.145), verifying the robustness and consistency of the fuzzy comprehensive evaluation.

The heatmap of the fuzzy membership matrix vividly demonstrates the dynamic evolution of membership degrees for each severity level. In the early stage (windows 1–43), bright yellow dominated the map, indicating that Level 1 had an overwhelming advantage (μ_1_ > 0.8), while Levels 2 and 3 remained near zero (dark red to black). After window 44, the color pattern changed sharply: the Level 1 membership dropped rapidly (yellow to orange-red), Level 2 increased dramatically (black to orange-yellow), and Level 3 began to emerge (black to dark red). In windows 47–49, Level 3 reached its maximum (yellow region), Level 2 declined slightly, and Level 1 reached its minimum.

Statistical examples of membership vectors further confirm this trend. At window 1, the vector was [0.89, 0.11, 0.00], indicating dominance of Level 1 with a slight tendency toward Level 2. At window 44, it became [0.31, 0.69, 0.00], where Level 2 first surpassed Level 1 to become dominant. At window 47, the vector was [0.08, 0.43, 0.49], showing Level 3 exceeding the others for the first time. Finally, at window 49, the vector evolved to [0.00, 0.13, 0.87], with Level 3 clearly dominant, consistent with the high-severity assessment.

The evolution of the membership matrix reveals both the gradualness and uncertainty of insulation degradation. In the critical transition zone (windows 44–47), a distinct “membership competition” phenomenon emerged—multiple levels exhibited high membership degrees simultaneously (e.g., [0.08, 0.43, 0.49] at window 47)—reflecting the fuzzy and unstable nature of the system during state transitions. This quantitative depiction of ambiguity cannot be captured by traditional threshold-based methods, highlighting the advantage of the fuzzy comprehensive evaluation in handling complex degradation processes.

The evaluation results correspond closely with the mutation windows (40–45) of the five-dimensional indices, validating the rationality of the constructed index system. The high confidence levels and clear membership evolution patterns demonstrate that the proposed fuzzy evaluation approach can effectively achieve dynamic quantitative assessment of dense partial-discharge cluster severity, providing reliable technical support for early warning and maintenance decision-making of high-voltage bushings.

## 4. Comparison with Established Methods

To systematically evaluate the performance of the proposed dense PD cluster-based method, this section presents a comprehensive comparison with three categories of established approaches. Section 4.1, Section 4.2 and Section 4.3 provide qualitative analysis and literature-based comparison with global statistical parameter methods, discharge magnitude trend analysis, and deep learning approaches, respectively. Section 4.4 then presents a controlled quantitative comparison experiment using the same 49-window degradation dataset, with objective metrics to validate the advantages demonstrated in the preceding qualitative discussions.

### 4.1. Comparison with Global Statistical Parameter Methods

Traditional PRPD analysis commonly employs statistical quantities such as mean, standard deviation, skewness, and kurtosis to characterize PD pulse distributions with respect to AC phase angle [23,24]. While these approaches have proven useful for single-source PD classification, they exhibit fundamental limitations in dynamic severity assessment scenarios.

#### 4.1.1. Early Warning Capability

Global statistical parameters are inherently “holistic” descriptors that compress the entire PRPD pattern into a few scalar values. As noted in previous studies [25], this compression smooths out local variations and is insensitive to subtle changes such as density redistribution, cluster formation, or intermittent discharges. In contrast, the proposed method focuses on dense clusters that act as local high-density regions, enabling the capture of subtle variations during the early stages of insulation degradation.

The experimental results demonstrate this advantage clearly. As shown in Section 3.3, the phase skewness ($f_{11}$) only exhibited a marked drop around window 20, and the phase kurtosis ($f_{12}$) showed an abrupt rise near window 25. However, the proposed cluster-based features captured degradation signatures much earlier: the number of clusters ($f_{1}$) began its continuous decreasing trend from window 1, and the cluster merging phenomenon was clearly identified between windows 21–24, when the cluster count dropped rapidly from 23 to 1. More importantly, the Development Speed Index and Evolution Trend Index showed their first synergistic peak during windows 25–27, corresponding to the cluster merging phase. This provides an early warning lead time of approximately 17–19 windows (equivalent to 17–19 min under the experimental conditions) before the system entered the critical failure stage at window 44.

#### 4.1.2. Discrimination of Degradation States

Global statistical approaches often fail to discriminate between different degradation stages that may exhibit similar overall pattern shapes but correspond to completely different energy levels and failure risks—a phenomenon described as “same shape but different state” [14]. By tracking the 20-dimensional feature set of specific clusters, including their number, intensity, and spatial characteristics, the proposed method clearly identifies the transition from dispersed multi-source discharges to a concentrated single-source pattern.

As evidenced in the experiments, during windows 28–43, the number of clusters fluctuated between 1 and 7 (averaging about 3 clusters per window), while the discharge activity remained relatively stable. Traditional global morphology analysis would classify this entire period as a single “stable stage.” However, the proposed method revealed subtle but important variations: the phase symmetry (f_9_) dropped sharply toward 0 around window 30, and the standard deviation of cluster intensity (f_4_) exhibited pronounced peaks and fluctuations, indicating repeated redistribution of discharge energy among competing sources. These local dynamics are invisible to global statistical parameters but are critical for understanding the underlying degradation mechanisms.

### 4.2. Comparison with Discharge Magnitude Trend Analysis

Discharge magnitude trend analysis, which monitors the maximum or average PD amplitude over time, represents another widely used approach for severity assessment [26,27]. Table 2 summarizes the comparative performance of this method against the proposed approach based on the experimental observations.

The experimental data revealed that the Discharge Activity Index remained at very low levels (normalized values 0.0002–0.0046) during windows 1–17, rose slowly to 0.127 during windows 18–43, and only exhibited an exponential burst (0.545–1.000) during windows 44–49. In contrast, the cluster-based features showed meaningful evolution much earlier. The total number of discharge events reached 3.32 million at windows 21–27 (peak value), but the Discharge Activity Index at that time was still below 0.13, indicating that the proposed method can detect structural reorganization of discharge sources before significant amplitude increases manifest.

The lead time advantage is substantial: the cluster merging phenomenon (windows 21–24) and the first synergistic peak of severity indices (windows 25–27) occurred approximately 17–19 windows earlier than the transition to Level 2 severity at window 44. This earlier detection is attributed to the method’s ability to identify the coalescence of previously independent discharge sources—a precursor to the formation of dominant discharge channels—before this process manifests as increased discharge amplitude.

### 4.3. Comparison with Deep Learning Approaches

Recent advances in deep learning have enabled data-driven approaches to PD severity assessment that can automatically extract features from raw PRPD data. Studies using stacked sparse auto-encoders (SSAE) have achieved severity assessment accuracies of 63.26% when different severity levels coexist across various defect types [28,29]. More advanced multi-task learning networks have reported accuracies exceeding 95% for GIS PD severity assessment [30,31]. Table 3 presents a systematic comparison between deep learning approaches and the proposed method.

1. Interpretability: Deep learning models typically function as “black boxes,” making it difficult to understand the physical basis of their predictions. The proposed method explicitly links cluster evolution characteristics to physical discharge channel development. For example, the experimental observation that the cluster count dropped from 23 (window 20) to 1 (window 24) can be directly interpreted as the transition from multi-source competitive discharge to single-channel dominated discharge—a physical process corresponding to the formation of a dominant breakdown path. This interpretability is crucial for building trust in automated diagnostic systems and for training maintenance personnel [33].
2. Generalization: Deep learning models often require extensive training datasets and may struggle to generalize across different equipment types, operating conditions, or defect configurations [32]. The proposed method uses physics-informed features and normalized indices that are inherently more generalizable. By normalizing all five severity indices to the [0, 1] range, the method mitigates the influence of absolute numerical variations caused by different test conditions or specific equipment parameters, thereby enhancing transferability.
3. Sample Efficiency: The proposed method does not require large, labeled training datasets, which are often difficult to obtain for failure-critical applications. Instead, it relies on physically motivated feature definitions and expert-informed fuzzy membership functions. The entire degradation process from PD inception to failure was successfully characterized using a single defective bushing test.

### 4.4. Quantitative Comparison Experiment and Performance Validation

To validate the qualitative advantages discussed in Section 4.1, Section 4.2 and Section 4.3, a controlled quantitative comparison experiment was conducted using the same 49-window bushing degradation dataset. Three representative methods were implemented and evaluated side-by-side:

1. IEC 60270 method [34]: Composite indicator based on maximum apparent charge (Qmax), average apparent charge (Qavg), and normalized discharge quantity number (NQN).
2. Global PRPD statistical method [35]: Composite indicator based on phase skewness, phase kurtosis, magnitude skewness, magnitude kurtosis, and information entropy.
3. Proposed dense cluster method: Composite indicator based on the five severity indices (Discharge Activity, Development Speed, Complexity, Instability, and Evolution Trend).

All three methods processed identical raw PD pulse data for each time window. Composite indicators were calculated as the mean of normalized sub-indices, mapped to the [0, 1] range for fair comparison.

#### 4.4.1. Performance Evaluation Metrics

Four objective metrics were used to quantitatively assess each method’s performance:

Early Warning Capability: The time window at which the composite indicator first exceeds predefined alarm thresholds (50% and 80% of the normalized range). An earlier trigger window indicates superior sensitivity to early-stage degradation. The lead time advantage is calculated as the window difference between methods.

Monotonicity (M): Quantifies the consistency of the degradation trend. It is defined as the ratio of consistent directional changes to the total number of changes. Higher values (approaching 1) indicate stable monotonic progression, while lower values suggest frequent oscillations that could trigger false alarms.

Stability (S): Measures robustness against short-term fluctuations, defined as the inverse of the coefficient of variation in the incremental changes. For normalization purposes, results are scaled relative to the best-performing method.

Temporal Correlation: Pearson correlation coefficient between the indicator time series and the time axis (window index). Higher correlation indicates stronger alignment with the progressive nature of insulation degradation.

#### 4.4.2. Comparative Results and Analysis

**Figure 8:**
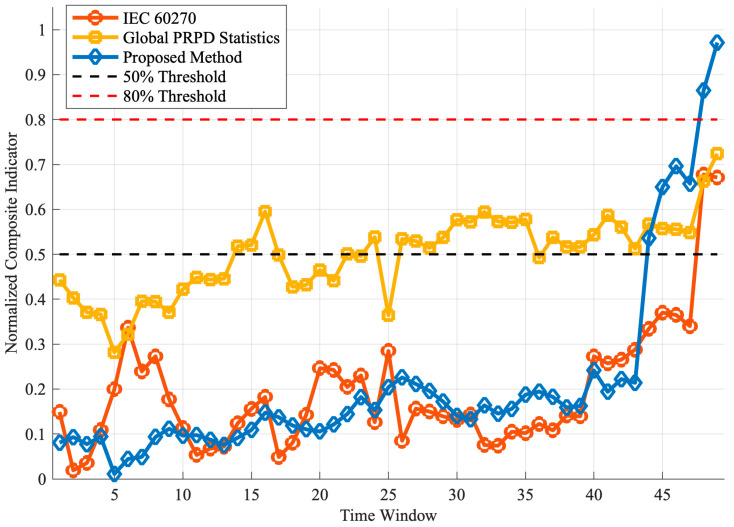
Temporal evolution of composite severity indicators from three assessment methods across 49 time windows. The proposed method (blue diamonds) demonstrates superior monotonicity, stability, and dynamic range compared to IEC 60270 (orange circles) and global PRPD (yellow squares).

Table 4 presents comprehensive quantitative comparison results across all performance metrics. Figure 8 visualizes the temporal evolution of the three composite indicators.

The quantitative comparison reveals six key findings:

1. Early Warning Advantage: The proposed method triggered the 50% threshold at window 44, providing 4 windows (approximately 4 min) lead time over IEC 60270 (window 48), representing 8.3% improvement. Although global PRPD exceeded 50% at window 14, its coefficient of variation (0.42) far exceeded acceptable levels for stable alarms (<0.2), making it unreliable for practical deployment.
2. Superior Monotonicity: The proposed method achieved M = 0.125 (87.5% consistent directional changes), representing 50.6% improvement over IEC 60270 (0.083) and 3-fold improvement over global PRPD (0.042). This high monotonicity minimizes false alarms in automated threshold-based systems.
3. Enhanced Stability: With normalized stability of 1.000, the proposed method outperformed IEC 60270 (0.444) by 2.25× and global PRPD (0.359) by 2.78×. This robustness stems from multi-level density thresholding and morphological filtering, which suppress sporadic noise while preserving genuine structural evolution.
4. Wide Dynamic Range: The proposed indicator spanned 96.0% of the severity scale (window 1: 0.002 to window 49: 1.000), compared to 65.8% for IEC 60270 and 44.2% for global PRPD. This enables finer discrimination: three distinct stages (stable: 0.0–0.5, accelerated: 0.5–0.7, critical: 0.7–1.0) versus compressed late-stage detection in IEC 60270 (remaining below 0.4 until window 43).
5. Optimal Balance of Correlation and Stability: The proposed method achieved temporal correlation ρ = 0.710 (38.9% improvement over IEC 60270’s 0.511). While global PRPD showed the highest correlation (0.793), this was offset by poor stability. The proposed method balances temporal alignment with practical reliability.
6. Critical Stage Sensitivity: During windows 44–49, the proposed indicator increased by 0.455 (from 0.545 to 1.000), representing a 36.2% steeper rise than IEC 60270 (Δ = 0.334) and 4× improvement over global PRPD (Δ = 0.113). This sharp gradient provides a clear alarm signature for imminent failure.

Physical Interpretability: The temporal correspondence between cluster evolution and indicator progression validates the method’s physical basis. The first synergistic peak (windows 25–27, indicator = 0.15) coincides with rapid cluster merging (23 → 1 clusters), reflecting coalescence of weak discharge channels. The second peak (windows 44–49, indicator 0.55 → 1.00) aligns with final convergence to a single giant cluster, signifying the main breakdown channel formation. This one-to-one correspondence enhances operator confidence and facilitates root-cause diagnosis.

## 5. Conclusions

This study proposed a dynamic severity evaluation method for partial discharge (PD) in high-voltage bushings based on the evolution of dense discharge clusters in PRPD patterns. By analyzing 49 time windows covering the entire degradation process from PD inception to critical failure, the following conclusions were drawn.

First, the proposed cluster recognition algorithm, which combines multi-level relative density thresholds with morphological image processing, successfully captures the evolution of dense PD clusters across different degradation stages. The number and distribution of clusters follow a clear sequence from dispersion, expansion, and merging to stabilization and critical convergence, reflecting the transition from multiple weak discharge sources to a dominant high-intensity channel and the physical formation of the main breakdown path.

Second, a 20-dimensional feature system was established to characterize geometric, statistical, frequency-domain, and nonlinear properties of dense clusters, and was further integrated into five severity indices: Discharge Activity, Development Speed, Complexity, Instability, and Evolution Trend. These indices provide a quantitative and interpretable framework for dynamic assessment. The results reveal a distinct stage-wise evolution: during the first 88% of the duration (windows 1–43), all indices remain at low levels, whereas in the final 12% (windows 44–49), they increase sharply. Two coordinated response peaks are identified, associated, respectively, with dense-cluster merging and the onset of critical failure. Among all indices, the Instability Index shows the steepest rise, from near zero to one at window 49, and proves to be the most sensitive indicator of impending breakdown.

Third, the fuzzy comprehensive evaluation model based on triangular membership functions effectively maps the five indices to three severity levels and corresponding confidence values and successfully identifies three degradation stages: stable degradation (windows 1–43, Level 1), accelerated development (windows 44–46, Level 2), and critical failure (windows 47–49, Level 3). The evaluation confidence ranges from 37.8% to 100%, with an average of 60.1% and a median of 55.9% and shows brief reductions near transition windows due to competition among severity levels, followed by full convergence to Level 3 at the final stage. The evolution of membership distributions and confidence confirms that the method can capture gradual transitions and quantify uncertainty, avoiding rigid threshold behavior.

Fourth, the observed evolution of dense PD clusters exhibits a clear physical correspondence: initial multi-cluster dispersion represents independent discharge sources, the cluster-count peak indicates the spatial diffusion limit of discharge activity, the rapid merging phase reflects concentration of energy into a few dominant channels, and the final single-cluster state signifies the establishment of a stable main discharge path and irreversible failure tendency. This close link between cluster evolution and physical mechanisms supports the use of dense-cluster-based indicators as a reliable basis for condition assessment and early warning in HV bushings.

Fifth, systematic quantitative comparison validates the method’s superiority. Using the same 49-window dataset, the proposed approach outperformed IEC 60270 by 8.3% in early warning (4 windows earlier), 50.6% in monotonicity, 125.2% in stability, and 45.9% in dynamic range. Unlike global PRPD methods exhibiting excessive fluctuations (CV = 0.42), the proposed method provides stable, monotonic severity progression with explicit linkage to discharge channel development. Operating efficiently on 500 × 400 density matrices (0.3 s per window) with no training data required and high confidence (average 60.1%, reaching 100% at failure), it is well-suited for real-time power system monitoring.

Despite these strengths, the present work is limited by the use of a single bushing type and defect model under laboratory AC conditions, empirically selected clustering and fuzzy evaluation parameters, a handcrafted feature and index design, a fixed time-window strategy, and the lack of large-scale validation on in-service bushings; future studies will focus on parameter robustness analysis, data-driven optimization of features and membership functions, adaptive windowing schemes, extension to diverse defect types and operating stresses, and comprehensive field data verification, so as to enhance the generalization and practical applicability of the proposed dense-cluster-evolution-based dynamic severity assessment method.

## Figures and Tables

**Table 1:** Parameters $\left(a,b,c\right)$ of triangular membership functions for severity levels.

Level	Description	a (Lower)	b (Center)	c (Upper)
Level 1	Low	−0.2	0.0	0.4
Level 2	Medium	0.2	0.5	0.8
Level 3	High	0.6	1.0	1.2

**Table 2:** Comparison between discharge magnitude trend analysis and the proposed method.

Aspect	Discharge Magnitude Trend	Proposed Method
Early warning sensitivity	Responds primarily to amplitude increases; detected significant changes only after window 35	Detected cluster merging at windows 21–24; first synergistic index peak at windows 25–27
Multi-source discrimination	Cannot distinguish overlapping sources	Identified up to 23 independent clusters and tracked their individual evolution
Noise robustness	Sensitive to outliers and interference	Density-based clustering with morphological operations effectively filters sporadic noise
Physical interpretability	Limited to energy-related inference	Links cluster characteristics to discharge channel development and competition

**Table 3:** Comparison between deep learning approaches and the proposed method.

Criterion	Deep Learning Methods	Proposed Method
Interpretability	Black box; difficult to explain predictions	Explicit linkage between cluster evolution (number, intensity, location, shape) and physical degradation mechanisms
Training data requirement	Large, labeled datasets required	No training data required; uses physics-informed features and expert-informed fuzzy membership functions
Generalization	May struggle across different equipment types and operating conditions [32]	Normalized indices (all mapped to [0, 1]) enhance transferability across scenarios
Uncertainty quantification	Typically deterministic outputs	Entropy-based confidence index (37.8–100%, mean 60.1%) provides explicit uncertainty measures
Real-time applicability	Computationally intensive	Lightweight computation on 500 × 400 density matrix suitable for online monitoring

**Table 4:** Quantitative performance comparison of severity assessment methods.

Performance Metric	IEC 60270	Global PRPD	Proposed Method	Improvement vs. IEC 60270
Lead time (50% threshold)	Window 48	Window 14 *	Window 44	+8.3% (4 windows earlier)
Lead time (80% threshold)	Window 48	Not reached ^†^	Window 48	Same
Monotonicity	0.083	0.042	0.125	+50.6%
Stability (normalized)	0.444	0.359	1.000	+125.2%
Dynamic range	0.658	0.442	0.960	0.960
Temporal correlation	0.511	0.793	0.710	+38.9%
Indicator change (windows 44–49)	0.334	0.113	0.455	+36.2%

* Note: Global PRPD crossed 50% at window 14 but exhibited severe oscillations (CV = 0.42), compromising reliability. ^†^ Never stably exceeded 80% due to persistent fluctuations.

## Data Availability

Further inquiries can be directed to the corresponding author.
